# Italian Version of the Cornell Assessment of Pediatric Delirium: Evaluation of the Scale Reliability and Ability to Detect Delirium Compared to Pediatric Intensive Care Unit Physicians Clinical Evaluation

**DOI:** 10.3389/fped.2022.894589

**Published:** 2022-05-18

**Authors:** Paola Claudia Fazio, Marco Daverio, Maristella Masola, Igor D’Angelo, Sara Frison, Cristina Zaggia, Silvio Simeone, Gianluca Pucciarelli, Dario Gregori, Rosanna Comoretto, Angela Amigoni

**Affiliations:** ^1^Pediatric Intensive Care Unit, Department of Woman’s and Child’s Health, University Hospital of Padova, Padua, Italy; ^2^Department of Woman’s and Child’s Health, University of Padua, Padua, Italy; ^3^Department of Clinical and Experimental Medicine, University “Magna Graecia,” Catanzaro, Italy; ^4^Department of Biomedicine and Prevention, University of Rome Tor Vergata, Rome, Italy; ^5^Unit of Biostatistics, Epidemiology and Public Health, Department of Cardiac, Thoracic, Vascular Sciences and Public Health, University of Padua, Padua, Italy; ^6^Department of Public Health and Pediatrics, University of Turin, Turin, Italy

**Keywords:** Cornell Assessment of Pediatric Delirium, CAPD, pediatric delirium, pediatric intensive care unit, PICU

## Abstract

**Background:**

Delirium is an acute brain dysfunction associated with increased length of hospitalization, mortality, and high healthcare costs especially in patients admitted to the pediatric intensive care unit (PICU). The Cornell Assessment of Pediatric Delirium (CAPD) is a screening tool for evaluating delirium in pediatric patients. This tool has already been used and validated in other languages but not in Italian.

**Objectives:**

To test the reliability of the Italian version of the CAPD to screen PICU patients for delirium and to assess the agreement between CAPD score and PICU physician clinical evaluation of delirium.

**Methods:**

Prospective double-blinded observational cohort study of patients admitted to a tertiary academic center PICU for at least 48 h from January 2020 to August 2021. We evaluated intra- and inter-rater agreement using the Intraclass Correlation Coefficient (ICC). The ability of the scale to detect delirium was evaluated by comparing the nurses’ CAPD assessments with the clinical evaluation of a PICU physician with expertise in analgosedation using the area under the ROC curve (AUC).

**Measurements and Main Results:**

Seventy patients were included in the study. The prevalence of pediatric delirium was 54% (38/70) when reported by a positive CAPD score and 21% (15/70) when diagnosed by the PICU physician. The CAPD showed high agreement levels both for the intra-rater (ICC 1 0.98, 95% CI: 0.97–0.99) and the inter-rater (ICC 2 0.93, 95% CI: 0.89–0.96) assessments. In patients with suspected delirium according to the CAPD scale, the observed sensitivity and specificity of the scale were 0.93 (95% CI: 0.68–1.00) and 0.56 (95% CI: 0.42–0.70), respectively. The AUC observed was 0.75 (95% CI: 0.66–0.8490).

**Conclusion:**

The Italian version of the CAPD seems a reliable tool for the identification of patients at high risk of developing delirium in pediatric critical care settings. Compared to the clinical evaluation of the PICU physician, the use of the CAPD scale avoids a possible underestimation of delirium in the pediatric population.

## Introduction

Delirium is a common and severe neuropsychiatric complication in critically ill patients defined by the Diagnostic and Statistical Manual of Mental Disorder V (DSM-V) as a disturbance of attention and awareness which develops over a short period of time from a patient’s baseline (1, 2). It may appear as hyperactive, hypoactive, and mixed subtypes. There is a large literature describing the incidence, duration, risk factors, subtypes, and outcomes of delirium in the adult population (3–5); however, the lack of use of a common diagnostic tool, the few prospective studies contributed the difficulty of interpreting the impact of the delirium on pediatric population (6, 7). Pediatric delirium has recently received increasing attention for the negative effects on critically ill children admitted to pediatric intensive care units (PICUs), among which a significantly increased length of hospitalization, mortality and high healthcare costs (8). According to a recent study, delirium incidence rates in the pediatric population are estimated to reach up to 57% of patients admitted to PICUs (9). Delirium in children can be difficult to recognize because its symptoms can fluctuate over hours and days and may be confused with those of other medical conditions (8, 10). The Cornell Assessment of Pediatric Delirium (CAPD) is a screening tool for the assessment of delirium in pediatric patients admitted to the PICU which demonstrated a good performance in children of all ages for the accurate and timely identification of delirium in this high-risk population. A recent position statement by the European Society of Paediatric and Neonatal Intensive Care (ESPNIC) recommended the use of CAPD as an instrument to assess pediatric delirium in critically ill infants and children (grade A of recommendation) (11) and its use has been implemented as a standard of care in a growing number of European centers. This tool has been translated and/or previously tested for reliability in different countries such as Japan, Portugal, Denmark, and Spain (9, 12–14). The CAPD was previously translated into Italian to guarantee linguistic equivalence to the original version (15), but its use in clinical practice has yet to be evaluated.

Therefore, the primary aim of this study was to analyze the reliability of the CAPD tool and the performance of each item of the scale. The secondary aim was to compare the CAPD results with the clinical assessment of delirium performed by PICU physicians.

## Materials and Methods

### Design and Setting

The study was set up as a single-center prospective double-blinded observational cohort data collection of patients admitted to the 10-bed PICU of the academic teaching University Hospital of Padova from January 2020 to August 2021. This is a mixed PICU which admits critically ill children with medical, surgical (both general and cardiac surgery), and traumatic diseases. This study was approved by the Ethics Committee of the University Hospital of Padova (CODE CESC 4792/AO/19 and CODE URC AOP1605, 10 October 2019).

### Study Population

The study enrolled pediatric patients less than 18 years old admitted to the PICU. All patients were included after a caregiver signed the informed consent. The following exclusion criteria were applied: (i) subjects whose parents were unavailable or unwilling to provide their consent; (ii) premature babies with a gestational age less than 37 weeks; (iii) subjects who were paralyzed, deeply sedated, or with a COMFORT Behavior Scale (CBS) score less than 11 (i.e., unarousable to verbal stimulation and therefore they could not be assessed for delirium); (iv) subjects with severe neurological diseases and with a Pediatric Cerebral Performance Category (PCPC) score more than 3 to reduce the risk for any bias during the assessment (16).

### The Cornell Assessment for Pediatric Delirium

The CAPD is an adaptation of the Pediatric Anesthesia Emergence Delirium (PAED) (7). The tool consists of eight questions aiming to assess critically ill children who are at risk of developing delirium, and it was designed to detect the symptoms of delirium. All questions correlate with DSM-V diagnostic domains and include psychomotor symptoms as well. Every question has a score from 0 to 4 points and a range from “never” to “always,” with a total score ranging from 0 to 32. A CAPD score of 9 or higher was considered as positive for the presence of delirium. The tool is associated with anchor points which indicate the development and behavior of children in different age groups.

### Study Procedures

In this study, we continued the CAPD psychometric validation process after the initial translation of the scale by Simeone et al. (15) (see Supplementary Table 1).

The assessment of CAPD scores was conducted by two clinical nurses (rater A and rater B) with different working experiences in the PICU (rater A with more than 2 years of experience in PICU, rater B with PICU experience between one and 2 years). The child’s bedside assessment was done as early as possible and when the CBS score was adequate.

The two nurses evaluated the patients using both the CBS and the Italian-CAPD:

1. Rater A performed a first and a second evaluation after a time lag of 2 min from the end of the first assessment for the intra-rater agreement;
2. Rater A and rater B performed the evaluations simultaneously in double-blind for the calculation of the inter-rater agreement.

The raters also collected data on the presence of parents, light, noise, and ongoing care activities. Each child was identified anonymously with a sequential three-digit numerical code. The results of the CAPD score were recorded in a paper Data Collection Form. All the files were collected by the nurse in charge of the study and inserted in an electronic database (Excel file) created for this study.

In this study, the final CAPD score was compared to the clinical assessment of delirium performed by two PICU physicians (MD and AA) with specific training in analgosedation who evaluated together and blinded from the nurses the patients while the nursing team was performing the CAPD score. The two physicians involved in the evaluation had more than 10 years of experience in the management of children in PICU and published more than 10 manuscripts on peer review journals on the analgosedation topic. In our setting, it was not possible to compare the CAPD score with a gold standard for delirium assessment, as it would require a child psychiatrist to confirm or reject the diagnosis of pediatric delirium (17). However, pediatric psychiatrists in our country do not have experience in PICU delirium and they are not usually involved in the care of these children. Therefore, the evaluation of delirium performed by PICU physician is considered the best delirium assessment to which we can aspire.

### Outcome Measures

The primary outcome measure of the present study was to evaluate the reliability of the CAPD scale defined as follows: (1) assessment of the intra- and inter-rater agreement of the CAPD scores between the two raters; (2) evaluation of the intra- and inter-rater agreement for each of the items of which the CAPD is composed.

The secondary outcome measure was the comparison between the ability of the tool in determining delirium and the pediatric delirium assessment performed by two PICU physicians.

### Sample Size

#### Assessment of the Intra- and Inter-Rater Agreement

The estimation problem refers to the evaluation of the concordance between the measures in terms of the Intraclass Correlation Coefficient (ICC). A moderate agreement between the measures is given by an ICC between 0.7 and 0.84. Different scenarios have been hypothesized for the calculation by varying the ICC from 0.7 to 0.9 following a step of 0.01. The approach used is that of the derivation of the ICC as suggested by Temel and Erdogan (18).

The calculation formula used is the following:

$$n=\frac{8\,Z_{1-\alpha /2}^{2}\,{\left(1-\rho_{p\,l\,a\,n}\right)}^{2}\,{\left[1+\left(k-1\right)\,\rho_{p\,l\,a\,n}\right]}^{2}}{k\,\left(k-1\right)\,W_{D}^{2}}$$

where, $Z_{1-\alpha /2}^{2}$ is the percentile of the normal standard associated with an alpha level of 0.05; ρ_*plan*_ is the ICC hypothesized to size the study; *k* is the number of measurements considered, in the specific case *k* = 2; *W*_*D*_ is the probability of the type II error in evaluating the estimated ICC as significantly different from zero.

As highlighted in the Supplementary Figure 1 is represented the accuracy of CAPD in predicting delirium using ROC curves, considering the PICU physician assessment as the best possible evaluation to be compared to. The black curve refers to the score cut-off of 9 while colored one’s report results for different score cut-off (from 8 to 15). The AUC for different scores are also reported. The best AUC could be found for the cut-off of 8 and 9 [0.755 (95% CI: 0.688–0.821) and 0.749 (95% CI: 0.656–0.841), respectively].

#### Assessment of the Sensitivity and Specificity of the Tool

In order to assess the sensitivity and specificity of CAPD tool, the sample size was determined using the area under the curve (AUC) estimation. The procedure is based on the optimization of the sample size determined by defining a specific margin of error d and a confidence level 1-α. Calculation has been performed using the approach proposed by Hajian-Tilaki (19). The formula applied is the following:

$$n=\frac{Z_{\alpha /2}^{2}\,V\,\left(A\,U\,C\right)}{d^{2}}$$

In the previous equation (AUC) can be estimated as:

$$V\,\left(A\,U\,C\right)=\left(0.0099\times e^{-\alpha^{2}/2}\right)\times \left(6\,\alpha^{2}+16\right)$$

where α = φ^−1^(*AUC*) × 1.414 and φ^−1^ is the inverse of the standardized cumulative distribution.

Different simulation scenarios have been defined for the calculation of the sample size by setting: (i) a 95% confidence level 1-α; (ii) an accuracy level d ranging from 0.08 to 0.1; (iii) an AUC value between 0.75 and 0.85, with a 0.01 step. The optimal sample size results for the various scenarios are presented in the Supplementary Figure 2. The results show that a sample size of 70 patients ensures a predictive ability of 0.8 with an error in the sample estimates *d* = 0.08.

Overall, a sample size of 70 subjects ensures the identification of both outcomes.

### Statistical Analysis

The descriptive analysis of the sample is reported using the median and the interquartile range (I–III quartile) for continuous variables given the non-parametric distribution and absolute numbers and percentages for categorical ones. The presence of statistically significant differences between two groups was assessed using the Wilcoxon–Kruskal–Wallis test for continuous variables and the χ^2^ test for categorical ones.

The intra- and inter-rater agreement was evaluated with the ICC [and its 95% confidence interval (CI)]. The sensitivity and specificity of the scale were evaluated by the calculation of the area under the curve (AUC) with the associated 95% CI.

The value of statistical significance considered as possible evidence of a difference between groups, after adjustment of the test values for test multiplicity according to the method by Benjamini and Hochberg, is set as *p* of 0.05 (20). The analyses were performed using R 4.1.1 (21) with pROC package (22).

## Results

### Cohort Descriptive Analysis

During the study period, 70 patients were enrolled with a total of 210 observations and corresponding CAPD scores reported. Table 1 reports the demographic and baseline characteristics of the overall population of patients included and the comparison of patients based on the presence of suspected Delirium (i.e., CAPD score ≥9) according to the first nurse evaluation. Overall, 40 patients (57%) were females, the median age was 7.11 months (IQR 1.98–52.73) and 11 patients (16%) were ex-premature. Forty-one patients (59%) have been evaluated while receiving mechanical ventilation.

**TABLE 1:** Characteristics and diagnosis of study subjects based on suspect of delirium (CAPD ≥ 9).

Characteristic	All study population (*N* = 70)	No suspect of delirium (*N* = 32)	Suspect of delirium (*N* = 38)	*p*-value
**Gender, % (*n*)**				0.013
Female	57% (40)	75% (24)	42% (16)	
Age, months, median (IQR)	7.11 (1.98-52.73)	11.13 (3.33-76.77)	6.13 (1.22-31.30)	0.278
**Age, categories, % (*n*)**				0.209
0–2 years	64% (45)	56% (18)	71% (27)	
3–5 years	11% (8)	9% (3)	13% (5)	
6–12 years	10% (7)	19% (6)	3% (1)	
13–17 years	14% (10)	16% (5)	13% (5)	
Prematurity, % (*n*)	16% (11)	12% (4)	18% (7)	0.498
PIM III at admission, median (IQR)	2.51 (1.14–5.42)	3.61 (1.20–5.94)	1.96 (1.02–4.60)	0.305
**Primary diagnoses, % (*n*)**				0.036
Cardiological disease	16% (11)	28% (9)	5% (2)	
Surgical	31% (22)	19% (6)	42% (16)	
Digestive	4% (3)	9% (3)	0% (0)	
Infective/inflammatory	4% (3)	3% (1)	5% (2)	
Neurological pathology	9% (6)	3% (1)	13% (5)	
Respiratory insufficiency	19% (13)	19% (6)	18% (7)	
Shock	6% (4)	3% (1)	8% (3)	
Polytrauma	1% (1)	0% (0)	3% (1)	
Other	10% (7)	16% (5)	5% (2)	
**Respiratory support, % (*n*)**				0.314
Non-invasive MV	20% (14)	28% (9)	13% (5)	
Invasive MV	59% (41)	47% (15)	68% (26)	
Length of ventilation (hours), median (IQR)	48. (0–138)	0 (0–78)	70 (23–191)	0.010
**Use of sedation, % (*n*)**				
Midazolam	49% (25)	19% (6)	50% (19)	0.009
Opiates	49% (34)	22% (7)	71% (27)	0.007
Ketamine	13% (9)	0% (0)	24% (9)	0.009
Analgosedation weaning, % (*n*)	43% (30)	28% (9)	55% (21)	0.036
Development of delirium*, % (*n*)	21% (15)	3% (1)	37% (14)	0.007

°Patients receiving sedation and drugs type at time of CAPD assessment. *Prevalence of delirium according to physicians’ evaluations.IQR, interquartile range; PIM III, Pediatric Index of Mortality Score III; MV, mechanical ventilation.

Patients with suspected delirium were more often male (58 vs 25%, *p* = 0.013) and evaluated during the analgosedation weaning process (55 vs 28%, *p* = 0.036). The median total duration of ventilation (considering both invasive and non-invasive mechanical ventilation) was significantly higher in patients with suspected delirium (70 h, IQR 23–191 vs 0 h, IQR 0–78; *p* = 0.0010). Moreover, suspected cases received more often a sedation with midazolam (*p* = 0.009), opiates (*p* = 0.007), and ketamine (*p* = 0.009).

### Intra- and Inter-Rater Agreement

Table 2 reports the concordance between the measures using the ICC. Considering the overall CAPD score, both intra-rater assessment (ICC 1 0.98, 95% CI: 0.97–0.99) and inter-rater assessment (ICC 2 0.93, 95% CI: 0.89–0.96) showed high agreement levels. Considering single item scores, only high intra-rater ICC (ICC 1) and moderate-to-high inter-rater ICC (ICC 2) have been observed. For almost all items, an inter-rater ICC 2 between 0.70 and 0.90 have been detected, except for item 1 (eye contact) which was higher (0.91, 95% CI: 0.86–0.94) and for item 3 (awareness) and 8 (respond) where a moderate inter-rater agreement was showed (item 3: ICC 2 0.80, 95% CI: 0.70–0.87 and item 8: 0.70, 95% CI: 0.56–0.80).

**TABLE 2:** CAPD scoring (overall and single item), intra-(ICC 1) and inter-(ICC 2) rater agreement.

	Rater A (1)	Rater B (1)	Rater A (2)	ICC 1 (95% CI)	*p*-value	ICC 2 (95% CI)	*p*-value
Overall score	10.00 (3.00–20.00)	11.50 (3.00–20.00)	10.50 (3.00–20.00)	0.98 (0.97–0.99)	<0.001	0.93 (0.89–0.96)	<0.001
Item 1 (eye contact)	1.00 (0.00–3.00)	1.00 (0.00–3.00)	1.00 (0.00–2.00)	0.95 (0.92–0.97)	<0.001	0.91 (0.86–0.94)	<0.001
Item 2 (action)	1.00 (0.00–3.00)	1.00 (0.00–3.00)	1.50 (0.00–3.00)	0.95 (0.93–0.97)	<0.001	0.87 (0.80–0.92)	<0.001
Item 3 (awareness)	1.00 (0.00–3.00)	1.00 (0.00–3.00)	1.00 (0.00–3.00)	0.94 (0.90–0.96)	<0.001	0.80 (0.70–0.87)	<0.001
Item 4 (communicate)	1.00 (0.00–3.00)	2.00 (0.00–4.00)	2.00 (0.00–3.00)	0.96 (0.94–0.98)	<0.001	0.85 (0.77–0.90)	<0.001
Item 5 (restless)	2.00 (1.00–2.00)	1.50 (1.00–2.00)	1.00 (1.00–2.75)	0.90 (0.85–0.94)	<0.001	0.85 (0.76–0.90)	<0.001
Item 6 (inconsolable)	1.00 (0.00–2.00)	1.00 (0.00–2.00)	1.00 (0.00–2.00)	0.93 (0.89–0.96)	<0.001	0.84 (0.75–0.90)	<0.001
Item 7 (underactive)	1.00 (0.00–2.00)	1.00 (0.00–1.75)	0.50 (0.00–2.00)	0.92 (0.87–0.95)	<0.001	0.89 (0.83–0.93)	<0.001
Item 8 (respond)	1.00 (0.00–2.00)	1.00 (0.00–2.00)	1.00 (0.00–2.00)	0.88 (0.81–0.92)	<0.001	0.70 (0.56–0.80)	<0.001

*ICC 1 = Intraclass Correlation Coefficient intra-rater (rater A at time 1 and rater A at time 2); ICC 2, Intraclass Correlation Coefficient inter-rater (rater A and operator B at time 1); CI, confidence interval.*

### Comparison of the Cornell Assessment of Pediatric Delirium Scores With the Pediatric Intensive Care Unit Physician Assessment

About half of the study cohort has been identified as cases of suspected delirium (*n* = 38, 54%) using the CAPD score, while the prevalence of pediatric delirium in our cohort diagnosed by the clinical assessment was 21% (*n* = 15).

Overall, patients’ with delirium not detected by the PICU physician were significantly younger than the rest of the population (median age 4 months, IQR 0.5–10 vs 14 months, IQR 3–119, *p* = 0.003) and received more frequently more than two sedatives than the other patients (46 vs 35%).

Table 3 shows the sensitivity and specificity according to the different cut-off of CAPD scale. Using the original cut-off of 9 of the CAPD score to identify patients with suspected delirium, the observed sensitivity and specificity of the CAPD scale were 0.93 (95% CI: 0.68–1.00) and 0.56 (95% CI: 0.42–0.70). A cut-off value of 8 for the CAPD total score provided a sensitivity of 1.00 (95% CI: 0.78–1.00), a specificity of 0.51 (95% CI: 0.37–0.65), a PPV of 0.36 (95% CI: 0.22–0.52), an NPV of 1.00 (95% CI: 0.88–1.00). Instead at the other extreme, a cut-off of 15 showed a sensitivity of 0.67 (95% CI: 0.38–0.88), a specificity of 0.71 (95% CI: 0.57–0.82), a PPV of 0.38 (95% CI: 0.20–0.59), and lastly a NPV of 0.89 (95% CI: 0.75–0.96). As the CAPD score cut-off increased, emerged a parallel raise of the specificity against sensitivity which was reduced.

**TABLE 3:** Sensitivity, specificity, PPV, and NPV according to different cut-off of the CAPD scale.

Cut-off	Apparent prevalence	PICU physician prevalence	Sensitivity (95% CI)	Specificity (95% CI)	PPV (95% CI)	NPV (95% CI)
Original (≥9)	0.54 (0.42–0.66)	0.21 (0.13–0.33)	0.93 (0.68–1.00)	0.56 (0.42–0.70)	0.37 (0.22–0.54)	0.97 (0.84–1.00)
≥8	0.60 (0.48–0.72)	0.21 (0.13–0.33)	1.00 (0.78–1.00)	0.51 (0.37–0.65)	0.36 (0.22–0.52)	1.00 (0.88–1.00)
≥10	0.53 (0.41–0.65)	0.21 (0.13–0.33)	0.87 (0.60–0.98)	0.56 (0.42–0.70)	0.35 (0.20–0.53)	0.94 (0.80–0.99)
≥11	0.49 (0.36–0.61)	0.21 (0.13–0.33)	0.80 (0.52–0.96)	0.60 (0.46–0.73)	0.35 (0.20–0.54)	0.92 (0.78–0.98)
≥12	0.47 (0.35–0.59)	0.21 (0.13–0.33)	0.80 (0.52–0.96)	0.62 (0.48–0.75)	0.36 (0.20–0.55)	0.92 (0.78–0.98)
≥13	0.41 (0.30–0.54)	0.21 (0.13–0.33)	0.73 (0.45–0.92)	0.67 (0.53–0.79)	0.38 (0.21–0.58)	0.90 (0.77–0.97)
≥14	0.39 (0.27–0.51)	0.21 (0.13–0.33)	0.73 (0.45–0.92)	0.71 (0.57–0.82)	0.41 (0.22–0.61)	0.91 (0.78–0.97)
≥15	0.37 (0.26–0.50)	0.21 (0.13–0.33)	0.67 (0.38–0.88)	0.71 (0.57–0.82)	0.38 (0.20–0.59)	0.89 (0.75–0.96)

*PPV, positive predictive value; NPV, negative predictive value; CI, confidence interval.*

**FIGURE 1:**
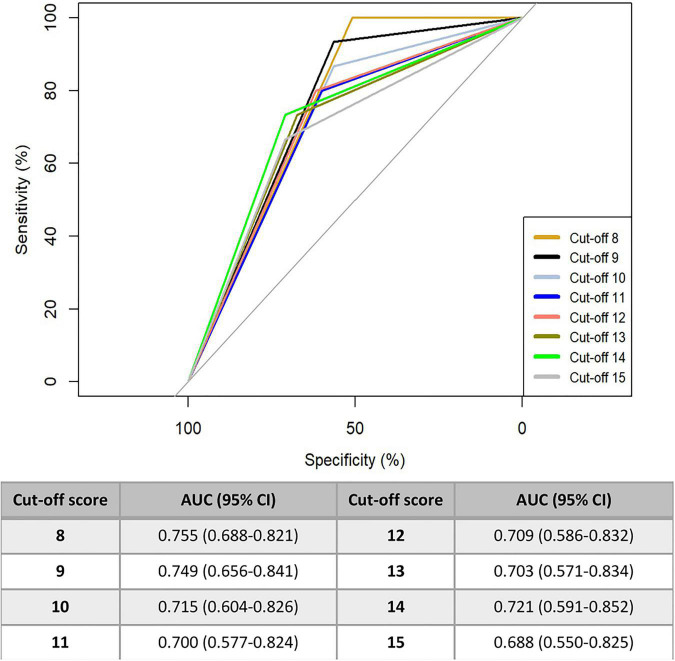
AUC for ROC curves based on different cut-off scores of CAPD tool.

In Figure 1 is represented the accuracy of CAPD in predicting delirium using ROC curves, considering the PICU physician assessment as the best possible evaluation to be compared to. The black curve refers to the score cut-off of 9 while colored one’s report results for different score cut-off (from 8 to 15). The AUC for different scores are also reported. The best AUC could be found for the cut-off of 8 and 9 [0.755 (95% CI: 0.688–0.821) and 0.749 (95% CI: 0.656–0.841), respectively].

## Discussion

The present study describes the cross-cultural adaptation of the CAPD scale from English to Italian and highlights a good reliability of this tool and a possible underestimation of the delirium prevalence when it is evaluated clinically by PICU physicians. Our results suggest that the Italian version of the CAPD scale shows a moderate to high intra- and inter-rater agreement for all items, as according to the original CAPD study (23).

The overall prevalence of delirium was 54% according to the CAPD score screening and 21% as per the clinical evaluation of the PICU physician. Current literature describes the delirium as a frequent complication of critical illness in childhood, with a point prevalence reported up to 57% (11, 24). The prevalence of delirium as assessed by the two physicians in our population was comparable to the delirium rate reported in the original CAPD validation study (i.e., 20.6%), but lower compared with other studies which included a higher percentage of children with delirium (11, 23–26). The underestimation of the phenomenon observed in our study could be due to the physicians performing the assessment, as they were not experienced psychiatrists, as it happens instead in other European regions. In fact, in our setting, psychiatrists do not have experience in PICU delirium and they are not usually involved in the management of critically ill patients affected by this disease. Despite the large experience and expertise in analgosedation, the two PICU physicians without the support of the CAPD may have misdiagnosed some of the patients leading to a possible underrating of the real delirium prevalence. The CAPD is a tool that does not aim to diagnose delirium, but to guide physicians to recognize the symptoms of delirium and to treat early these patients. Furthermore, patients with suspected delirium who were not detected by the PICU physicians were younger and more sedated than the other patients and their diagnosis could have been dismissed by intensivists performing non-standardized assessments. These patients could be suffering hypoactive delirium which has been previously reported as being the most frequent delirium subtype and more difficult to diagnose (8, 27, 28). This issue underlines the need for a screening program training on delirium and its risk factors within the PICU staff which should involve a multidisciplinary team composed of PICU nurses, physicians, and psychiatrists.

It is interesting to note that we found the highest prevalence of delirium in children requiring ventilation and with a higher need of midazolam, opiates, and ketamine. This finding may mirror a possible higher severity of illness in this sub-group. However, it should be noted that PIM III score has been assessed only at PICU admission but not at the moment of the CAPD evaluation; therefore, despite the similar PIM III values at arrival, we cannot exclude that they were significantly different at the time of the CAPD evaluation.

Intra- and inter-rater agreement analysis shows good results, reporting ICC above 0.70 both overall and for single items. Item 3 (awareness) and item 8 (respond) demonstrated the lowest inter-rater reliability with a moderate intraclass correlation (ICC 0.87 and 0.70, respectively) which was confirmed also with the lowest intra-rater agreement for item 8 (ICC 0.88). Awareness of the surroundings is difficult to determine in critically ill children, while the response time to interaction can be influenced by countless factors. However, these two values are still above the accepted threshold for defining a good agreement between the measures (i.e., ≥0.7 ICC). Nevertheless, improving the agreement for these questions may be an area of clinical investigation in future studies. Conversely, in the Japanese study by Hoshino, item 6 (inconsolable) and item 7 (underactive) showed a low inter-rater correlation, 0.67 and 0.69, respectively. This could be due both to the different measure used (Cohen’s *k*) and to the use of different exclusion criteria. In fact, we excluded children with severe neurological disorders to reduce further biases at the time of CAPD assessment. However, it is also important to underline that the inter-rater correlation was overall high, despite the different level of working experience of the evaluating nurses, demonstrating a good reliability of the scale.

Considering the CAPD accuracy using the AUC measurement, the Italian version demonstrated an optimal scoring cut-off point of 8, showing an area under the curve of 0.755 (95% CI: 0.688–0.821), while the AUC for the cut-off score of 9 is 0.749 (0.656–0.841). The cut-off value of 9 of the CAPD (usually used to discriminate patients at risk from those not at risk of delirium), showed a good balance between the sensitivity of the scale (which was very high, 93%) and its specificity (56%), maintaining a good false negative screen, in comparison to the other versions previously created both in English and in Japanese (23, 24). However, the cut-off point of 8 shows an even greater sensitivity, but with a further decrease in specificity (100 and 51%, respectively). Overall, CAPD appears to be an excellent screening instrument for assessing the risk of developing delirium, but it cannot be used alone as the only tool for the diagnosis of this disorder.

This study has several limitations that should be acknowledged. First, the abovementioned difference in delirium prevalence detected by the CAPD tool and the clinical evaluation can be explained by the fact that, in our setting, it was not possible to involve a psychiatrist in delirium evaluation and the assessment was performed by the PICU physician without the support of a validated tool. Indeed, the diagnosis of delirium may sometimes be difficult especially for the hypoactive subtype of patients. Furthermore, the study was conducted in a single center, possibly limiting the external validity of our results to other Italian PICUs.

## Conclusion

The Italian version of the CAPD showed a good intra- and inter-rater reliability and a high sensitivity for the detection of delirium in PICU. CAPD should be used as a screening tool to early identify patients with a high risk of developing delirium in pediatric critical care settings in order to avoid a possible underestimation of delirium in this population. We believe this translated version of the original scale can be applied by healthcare providers in Italy. Further studies would be helpful to confirm the reliability and to explore the validity of this translated version in other Italian PICUs.

## Data Availability Statement

The raw data supporting the conclusions of this article will be made available by the authors, without undue reservation.

## Ethics Statement

The studies involving human participants were reviewed and approved by the Ethics Committee of the University Hospital of Padova (CODE CESC 4792/AO/19 and CODE URC AOP1605, 10 October 2019). Written informed consent to participate in this study was provided by the participants’ legal guardian/next of kin.

## Author Contributions

All authors contributed to the study conception and design. PF, ID’A, MM, and SF performed the material preparation and data collection. PF, MD, and AA wrote the first draft of the manuscript. RC and DG performed the data analysis. All authors commented on previous versions of the manuscript and, read and approved the final manuscript.

## Conflict of Interest

The authors declare that the research was conducted in the absence of any commercial or financial relationships that could be construed as a potential conflict of interest.

## Publisher’s Note

All claims expressed in this article are solely those of the authors and do not necessarily represent those of their affiliated organizations, or those of the publisher, the editors and the reviewers. Any product that may be evaluated in this article, or claim that may be made by its manufacturer, is not guaranteed or endorsed by the publisher.

## Supplementary Material

Supplementary Figure 1 Sample size estimated for different ICC values (alpha = 0.05, beta = 0.2).

Supplementary Figure 2 Sample size for AUC and precision, confidence level 0.95.
