# Supplementary material for: Italian Version of the Cornell Assessment of Pediatric Delirium: Evaluation of the Scale Reliability and Ability to Detect Delirium Compared to Pediatric Intensive Care Unit Physicians Clinical Evaluation
Source: Front Pediatr. 2022 May 18;10:894589. doi: 10.3389/fped.2022.894589 (PMC9157792; doi:10.3389/fped.2022.894589)
Supplement: Supplementary file 1 [file Table_1.DOCX]

|  | Mai  4 | Raramente  3 | Qualche volta  2 | Spesso  1 | Sempre  0 | Score |
| --- | --- | --- | --- | --- | --- | --- |
| 1) Il bambino mantiene contatto visivo con il care-giver? |  |  |  |  |  |  |
| 2) Le azioni del bambino indicano intenzionalità? |  |  |  |  |  |  |
| 3) Il bambino è consapevole di chi gli sta intorno? |  |  |  |  |  |  |
| 4) Il bambino comunica le proprie necessità e desideri? |  |  |  |  |  |  |
|  | Mai  0 | Raramente  1 | Qualche volta  2 | Spesso  3 | Sempre  4 | Score |
| 5) Il bambino è irrequieto? |  |  |  |  |  |  |
| 6) Il bambino è inconsolabile? |  |  |  |  |  |  |
| 7) Il bambino è ipoattivo con rari movimenti durante la veglia? |  |  |  |  |  |  |
| 8) Il bambino necessita di molto tempo prima di rispondere alle interazioni? |  |  |  |  |  |  |
|  |  |  |  |  | TOTALE |  |

Cornell Assessment of Pediatric Delirium (CAPD) - Versione Italiana

Developmental Anchor Points For Youngest Patients - Versione Italiana

|  | Neonato | 4 settimane | 6 settimane | 8 settimane | 28 settimane | 1 anno | 2 anni |
| --- | --- | --- | --- | --- | --- | --- | --- |
| 1. Il bambino segue lo sguardo del caregiver? | Fissa il volto | Sostiene brevemente lo sguardo  Segue a 90 gradi | Sostiene lo sguardo | Segue un oggetto in movimento/caregiver per meta della loro traiettoria, osserva la mano del’esaminatore contenente l’oggetto, focalizza l’attenzione | Sostiene lo sguardo.Preferisce i genitori. Guarda chi parla | Sostiene lo sguardo.Preferisce i genitori. Guarda chi parla | Sostiene lo sguardo.Preferisce i genitori. Guarda chi parla |
| 2. Le azioni del bambino sono propositive? | Muove la testa lateralmente dominato dai riflessi primitivi | Cerca Di Raggiunge re  (con una certa mancanza di coordinazione) | Cerca di raggiungere | Movimenti simmetrici,passivamente prende gli oggetti che gli vengono porti | Raggiunge con movimento fluido e coordinato | Raggiunge e manipola gli oggetti,cerca di cambiare posizione e se mobile può tentare di alzarsi | Raggiunge e manipola gli oggetti,cerca di cambiare posizione e se mobile può tentare di alzarsi e camminare |
| 3. Il bambino è consapevole di ciò che lo circonda? | Calmo/tranquillo quando sveglio | Attento quando sveglio  Si gira verso la voce del caregiver primario  Può girarsi a sentire l’odore del caregiver primario | Aumenta il tempo in cui e’ sveglio ed attento  Si gira verso la voce del caregiver primario  Può girarsi a sentire l’odore del caregiver primario | Gli si illumina la faccia, sorride, “tuba” o fa smorfie in risposta ad annuimenti con la testa o al suono di una campanella | Preferisce fortemente la madre e poi gli altri familiari. Distingue oggetti nuovi da quelli a lui familiari | Preferisce i genitori rispetto ad altri familiari, si irrita quando è separato da loro. Confortato da oggetti familiari come una coperta preferita od un pupazzo | Preferisce i genitori rispetto ad altri familiari, si irrita quando è separato da loro. Confortato da oggetti familiari come una coperta preferita od un pupazzo |
| 4. Il bambino comunica i suoi bisogni e quello che vuole? | Piange quando ha fame o è a disagio | Piange quando ha fame o è a disagio | Piange quando ha fame o è a disagio | Piange quando ha fame o è a disagio | Vocalizza/indica I suoi bisogni, es.:fame, disagio, curiosità per oggetti o per ciò che lo circonda | Usa singole parole o segni | Frasi composte da 2-3 parole. Puo’ indicare i suoi bisogni igienici, nomina se stesso o “me” |
| 5. Il bambino è irrequieto? | Non rimane attento a lungo quando sveglio | Non riesce a rimanere calmo | Non riesce a rimanere calmo | Non riesce a rimanere calmo | Non riesce a rimanere calmo | Non riesce a rimanere calmo | Non riesce a rimanere calmo |
| 6. il bambino e’ inconsolabile? | Non calmato dal cullare dei genitori, dal canto, dall’alimentazione e da altre azioni confortanti | Non calmato dal cullare dei genitori, dal canto, dal cibo e altre azioni confortanti | Non calmato dal cullare dei genitori, dal canto, dal cibo e altre azioni confortanti | Non calmato dal cullare dei genitori, dal canto, dal cibo e altre azioni confortanti | Non si calma con i soliti modi, es.: con il canto, o il parlargli, col prenderlo in braccio | Non si calma con i soliti modi, es.: con il canto, o il parlargli, leggendogli o col prenderlo in braccio | Non si calma con i soliti modi, es.: con il canto, o il parlargli, col prenderlo in braccio, leggendo (Può essere capriccioso, ma dovrebbe calmarsi) |
| 7. Il bambino è poco attiVo-veramente pochi movimenti mentre è sveglio? | Pochi o nessun movimento seguiti da un rilassamento con riflessi primitivi  (il bambino dovrebbe dormire tranquillo la maggior parte del tempo) | Pochi o nessun tentavo di raggiungere, calci ed afferra (può essere anocra scoordinato) | Pochi o nessuno, calci ed afferra (può essere Piu coordinato) | Poca o nessuna intenzionalita’ nel raggiungere ed afferrare le cose, nel controllare la testa e movimenti degli arti, cosi come nell’ allontanare cose spiacevoli | Poca o nessuna intenzionalita’ nel raggiungimento di cose,nell’afferrarle, nel girarsi nel letto ed allontanare via le cose  spiacevoli | Poco o nessun gioco,pochi sforzi per sedersi e tirarsi su, e,se mobile, pochi sforzi per gattonare o aggrapparsi sui mobili o camminare | Poco o nessun progresso verso giochi piu elaborati, sforzi per sedersi, e , se in grado di stare in piedi, per camminare o saltare |
| 8. Al bambino occorre molto tempo per rispondere alle interazioni? | Non fa suoni o riflessi attivi come previsto (afferrare,succhiare, RIFLESSO DI MORO) | Non fa suoni o riflessi attivi come previsto (afferrare,succhiare, RIFLESSO DI MORO) | Non scalcia e non piange in risposta a stimoli spiacevoli | Non ‘tuba” sorride o si concentra con lo sguardo in risposta alle interazioni | Non balbetta o sorride/ride nelle interazioni sociali (neanche rifiuto attivo di una relazione) | Non segue semplici indicazione. Se parla non si impegna in un dialogo con semplici parole o gergo | Non segue semplici comandi verbali articolati in 1-2 sequenze. Se parla, non si impegna in dialoghi leggermente più complessi |
