# Supplementary figures and images for: Italian Version of the Cornell Assessment of Pediatric Delirium: Evaluation of the Scale Reliability and Ability to Detect Delirium Compared to Pediatric Intensive Care Unit Physicians Clinical Evaluation
Source: Front Pediatr. 2022 May 18;10:894589. doi: 10.3389/fped.2022.894589 (PMC9157792; doi:10.3389/fped.2022.894589)

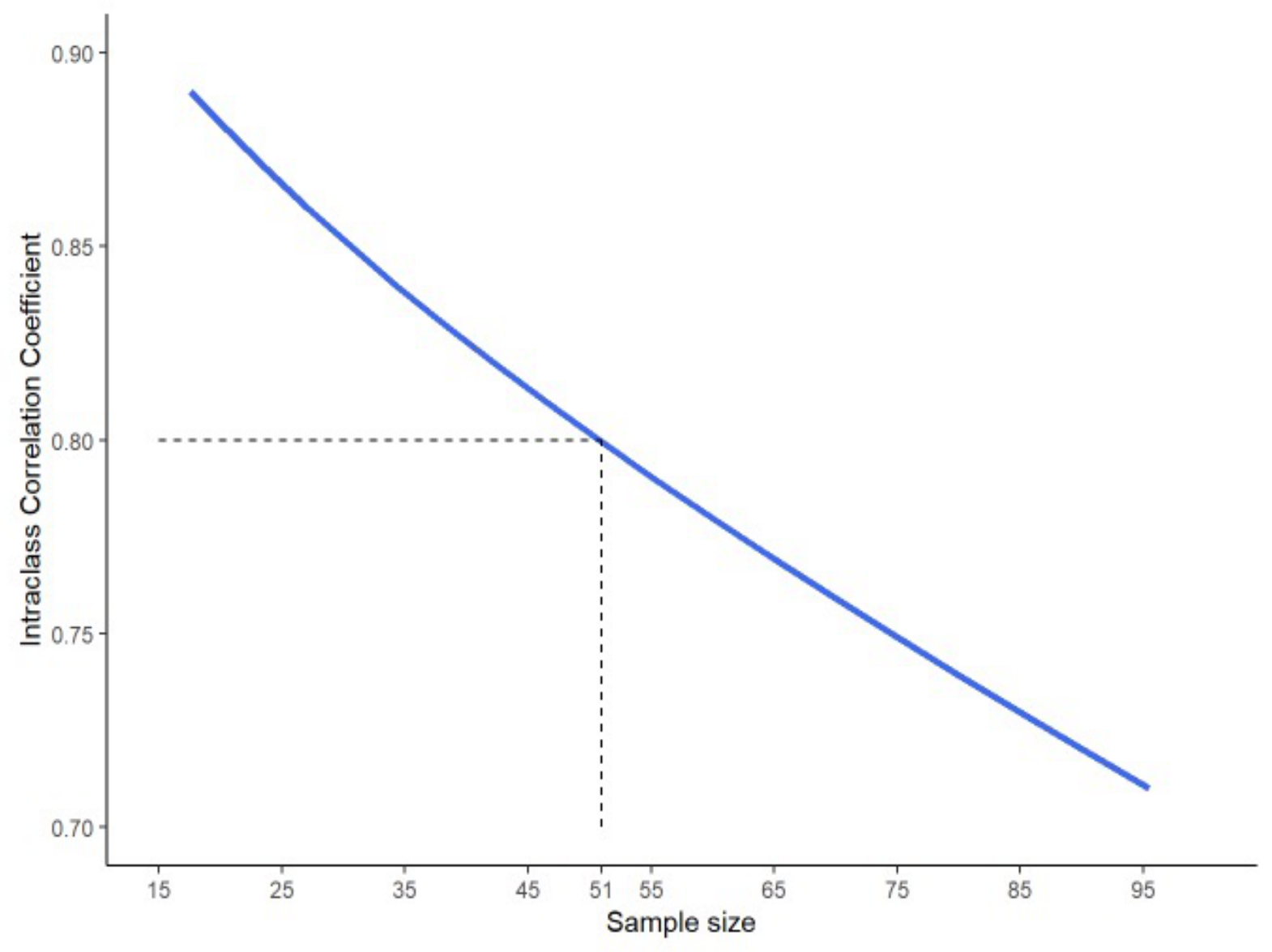

Supplement: Supplementary Figure 1 — Sample size estimated for different ICC values (alpha = 0.05, beta = 0.2). [file Image_1.TIFF]

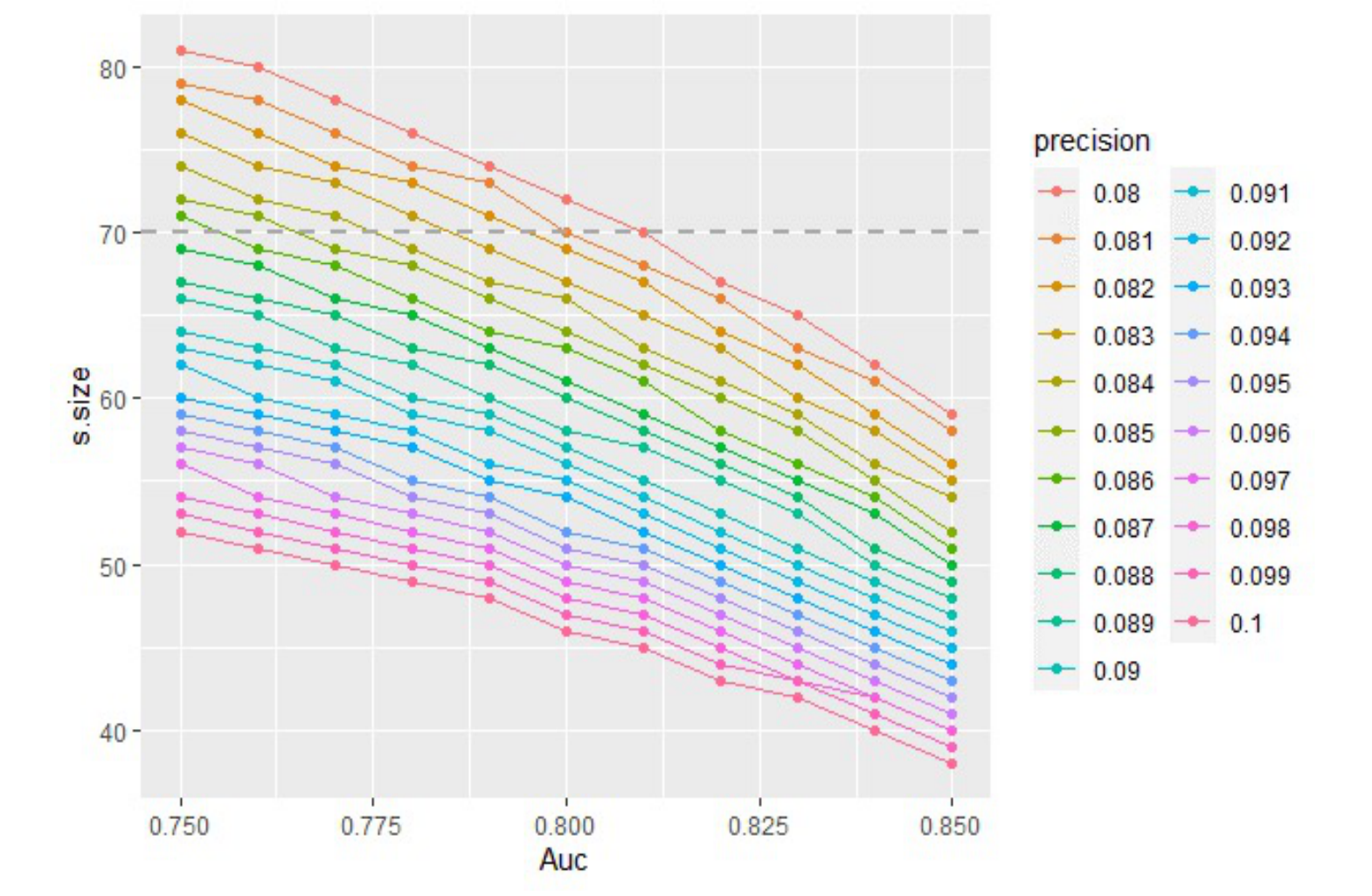

Supplement: Supplementary Figure 2 — Sample size for AUC and precision, confidence level 0.95. [file Image_2.TIFF]
